# Evaluation of the Temporal Characteristics of Ultrafast Imaging Methods Using Continuous Chirped Pulse Illumination

**DOI:** 10.3390/s25102957

**Published:** 2025-05-08

**Authors:** Yahui Li, Hang Li, Wanyi Du, Chao Ji, Kai He, Guilong Gao, Jinshou Tian

**Affiliations:** 1 State Key Laboratory of Ultrafast Optical Science and Technology, Xi’an Institute of Optics and Precision Mechanics, Xi’an 710119, China; liyahui@opt.ac.cn (Y.L.); duwanyi@opt.ac.cn (W.D.); jichao2017@opt.ac.cn (C.J.); hekai@opt.ac.cn (K.H.); gaoguilong@opt.ac.cn (G.G.); tianjs@opt.ac.cn (J.T.); 2 Collaborative Innovation Center of Extreme Optics, Shanxi University, Taiyuan 030006, China

**Keywords:** ultrafast imaging, single-shot imaging, active chirped pulse illumination, temporal characteristics

## Abstract

Ultrafast imaging based on chirped pulse illumination has opened new frontiers, offering a frame rate beyond 1 Tfps for the acquisition of multiple frames in a single–shot. However, the temporal resolving capability is implicitly influenced by parameters in stages of pulse illumination and data acquisition. This study delivers a mathematical model to produce a precise investigation, sorting the dominating factors, including the illumination pulse’s bandwidth $\lambda_{FWHM}$, dispersive propagation length *z*, and framing module’s spectral resolution Δλ. For a different $\lambda_{FWHM}$, *z* has a lower bound to ensure the covered signal is resolved; meanwhile, the time resolution decreases with a larger *z*. Frame extraction with a narrower Δλ leads to a higher time resolution; however, Δλ must be broad enough for a reasonable signal-to-noise ratio. The theoretical and experimental approaches to evaluate temporal characteristics are discussed, enabling a precise quantitative determination for the community to produce, use, and exploit single-shot ultrafast imaging systems.

## 1. Introduction

Ultrafast imaging techniques pave the way to the studies of dynamic phenomena beyond the temporal scale of nanoseconds [1,2,3,4,5,6], which are of great interest in diverse scientific research and industrial applications. When femtosecond time resolution is pursued, the pump-probe method is the golden standard [7,8]. However, it is not applicable for non-repetitive dynamic events, such as explosions [9], femtosecond laser ablation [10,11], and physical processes in semiconductors [12]. This promotes the development of single-shot ultrafast imaging techniques.

Several single-shot femtosecond photography approaches emerge to achieve multiple frame acquisition for a once-occurred dynamic event, including sequentially timed all-optical mapping photography (STAMP) [13], STAMP’s variant utilizing spectral filtering (STAMP-SF) [14,15], frequency recognition algorithm for multiple exposures (FRAME) [16,17], compressed ultrafast spectral–temporal photography (CUST) [18], and swept coded aperture real-time femto-photography (SCARF) [19]. The principle includes encoding of ultrafast illumination pulses spectrally or spatially and separation of time-resolved frames optically or computationally.

For STAMP and FRAME, a train of daughter pulses is needed to probe the target; individual daughter pulses carry different transient information. It is apparent that the exposure time, frame rate, and frame number are determined by the daughter pulses’ duration, interval, and numbers, respectively. The temporal properties, including exposure time and frame rate of STAMP, have been discussed and optimized in previous works [20]. For the methods using continuous chirped pulse illumination (STAMP-SF, CUST, and SCARF), the features of output frames are determined by not only the chirped pulse but also the spectrally resolving device. In [21], a full Fourier transform method was proposed to enhance the temporal resolution by measuring both the amplitude and phase of the chirped illumination probe.

However, the current works with continuous chirped pulse illumination lack a systematic analysis and an instructive guideline for the quantitative determination of temporal characteristics in a particular setup. It is desirable and essential to construct a feasible evaluation model to quantitatively analyze and optimize the temporal resolving capability, providing a more convincing interpretation of the captured results.

In this work, we investigate the temporal characteristics referring to the previous configurations using chirped pulse illumination, especially the time resolution, revealing the key factors in single-shot ultrafast photography using continuous chirped pulse illumination. Additionally, the procedure of the theoretical evaluation guideline and the potential experimental evaluation setup using an optical Kerr gate are discussed.

## 2. Methods and Theory

**Figure 1:**
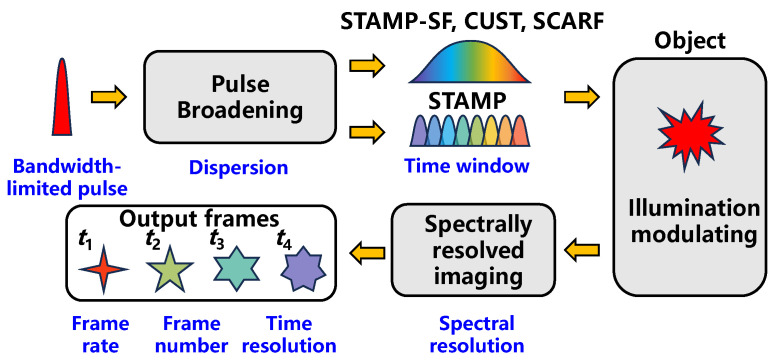
Schematic of ultrafast imaging methods based on chirped pulse illumination.

The process of ultrafast imaging based on chirped pulse illumination can be abstracted into three stages, as shown in Figure 1. During the pulse broadening stage, incident pulses are stretched into chirped pulses with a longer duration, which determines the observing time window $T_{window}$. For STAMP, the chirped pulse is chopped into a series of daughter pulses before illumination. For STAMP-SF, CUST, and SCARF, the chirped pulse is directly incident on targets. For the second stage, the object’s time-varying information is mapped to individual wavelengths. In the third stage, time-resolved frames are extracted using spectral imaging systems.

In the continuous chirped pulse case, the frame rate and frame number depend on the spectral interval between adjacent frames and the total number of spectral images. The time resolution is determined by the spectral resolution Δλ, chirped pulse’s bandwidth $\lambda_{FWHM}$, and dispersive properties. To evaluate the temporal properties quantitatively, the spectrum–time mapping model is constructed based on the physical data acquisition model, and the evaluation criteria are set for synthetic signals following sine functions.

### 2.1. Spectrum–Time Mapping Model

To evaluate the temporal characteristics quantitatively, the mathematical model of the spectrum–time mapping relationship of the chirped illumination is constructed. The electric field of a bandwidth-limited Gaussian pulse can be expressed as [22]

(1) $$E_{nc}\left(0,t\right)=\exp\left(-t^{2}/2\sigma_{0}^{2}\right)\;\exp\left(i\omega_{0}\;t\right)$$

where $\sigma_{0}$ is the standard deviation and $\omega_{0}$ is the center frequency. The full width at half maximum (FWHM) of the pulse intensity profile is $\tau_{0}=2\sqrt{\ln2}\sigma_{0}$. The corresponding expression of Equation (1) in the Fourier domain is

(2) $${\tilde{E}}_{nc}\left(0,\omega \right)=\int_{-\infty }^{\infty }E_{nc}\left(0,t\right)\exp\left(-i\omega t\right)dt$$

With the relationship $\int_{-\infty }^{\infty }\exp\left(-ax^{2}+bx\right)dx=\sqrt{\pi /a}\exp\left(b^{2}/4a\right)$, Equation (2) can be written as

(3) $${\tilde{E}}_{nc}\left(0,\omega \right)=\sqrt{2\pi }\sigma_{0}\exp\left[\frac{-{\left(\omega -\omega_{0}\right)}^{2}}{2/\sigma_{0}^{2}}\right]$$

Equation (3) reveals that the pulse power spectrum curve follows the Gaussian distribution with a FWHM of $\omega_{FWHM}=2\sqrt{\ln2}/\sigma_{0}$.

With the propagation in a dispersion medium at any point *z*, the field in the Fourier domain can be expressed as

(4) $${\tilde{E}}_{chirp}\left(z,\omega \right)={\tilde{E}}_{nc}\left(0,\omega \right)\exp\left[-ik\left(\omega \right)z\right]$$

where $k\left(\omega \right)$ is the wavenumber at ω. Expanding $k\left(\omega \right)$ in a Taylor series around the carrier frequency $\omega_{0}$,

(5) $$k\left(\omega \right)=k\left(\omega_{0}\right)+k^{\prime }\left(\omega -\omega_{0}\right)+\frac{1}{2}k^{\prime \prime }{\left(\omega -\omega_{0}\right)}^{2}+\frac{1}{6}k^{\prime \prime \prime }{\left(\omega -\omega_{0}\right)}^{3}+\ldots$$

Equation (4) can be rewritten as

(6) $${\tilde{E}}_{chirp}\left(z,\omega \right)=\sqrt{2\pi }\sigma_{0}\exp\left[-\left(\frac{\sigma_{0}^{2}}{2}+\frac{ik^{\prime \prime }z}{2}\right){\left(\omega -\omega_{0}\right)}^{2}-ik^{\prime }z\left(\omega -\omega_{0}\right)-ik\left(\omega_{0}\right)z\right]$$

where the cubic and higher-order terms in the expansion (Equation (5)) are excluded because they are negligible if $\omega_{FWHM}\ll \omega_{0}$ [22]. With the inverse Fourier transform of Equation (6), the expression of the chirped pulse in the temporal domain is

(7) $$\begin{array}{cc} E_{chirp}\left(z,t\right) & =\frac{1}{2\pi }\int_{-\infty }^{\infty }{\tilde{E}}_{chirp}\left(z,\omega \right)\exp\left(i\omega t\right)\;d\omega \end{array}$$

(8) $$\begin{array}{cc} \; & =\frac{\sigma_{0}}{\sqrt{2\pi }}\exp\left[i\omega_{0}t-ik\left(\omega_{0}\right)z\right] \end{array}$$

(9) $$\begin{array}{cc} \; & \times \int_{-\infty }^{\infty }\exp\left[-\left(\frac{\sigma_{0}^{2}}{2}+\frac{ik^{\prime \prime }z}{2}\right){\left(\omega -\omega_{0}\right)}^{2}+\left(it-ik^{\prime }z\right)\left(\omega -\omega_{0}\right)\right]d\omega \end{array}$$

which can be derived as

(10) $$E_{chirp}\left(z,t\right)=\sqrt{\frac{1}{1+iz/L_{D}}}\exp\left[i\omega_{0}t-ik\left(\omega_{0}\right)z\right]\exp\left[-\frac{{\left(t-z/v_{g}\right)}^{2}}{2\sigma_{0}^{2}\left(1+iz/L_{D}\right)}\right]$$

where $L_{D}=\sigma_{0}^{2}/k^{\prime \prime }$ is the dispersion length. The chirped pulse is broadened with a standard deviation $\sigma_{chirp}=\sigma_{0}\sqrt{1+{\left(z/L_{D}\right)}^{2}}$ and a FWHM $\tau_{chirp}=2\sqrt{\ln2}\sigma_{chirp}$. $v_{g}=1/k^{\prime }$ is the group velocity of the pulse envelope.

The chirped pulse in Equation (10) can be simplified as

(11) $$E_{chirp}\left(z,T\right)=\left|E_{chirp}\left(z,T\right)\right|\exp\left[i\phi (z,T)\right]$$

where $T=t-z/v_{g}$. The phase varies quadratically across the pulse at any *z*,

(12) $$\phi \left(z,T\right)=\frac{sgn\left(k^{\prime \prime }\right)\left(z/L_{D}\right)}{1+{\left(z/L_{D}\right)}^{2}}\frac{T^{2}}{2\sigma_{0}^{2}}+\frac{1}{2}\tan^{-1}\left[sgn\left(k^{\prime \prime }\right)\frac{z}{L_{D}}\right]$$

The time derivative of ϕ(z,T) is

(13) $$\delta \omega \left(T\right)=-\frac{\partial \phi }{\partial T}=\frac{sgn(k^{\prime \prime })\left(z/L_{D}\right)}{1+{\left(z/L_{D}\right)}^{2}}\frac{T}{\sigma_{0}^{2}}$$

where δω(T) is the frequency difference along the chirped pulse. $\omega =\omega_{0}+\delta \omega$ changes linearly with *T*, which is the chirped pulse spectrum–time mapping model.

**Figure 2:**
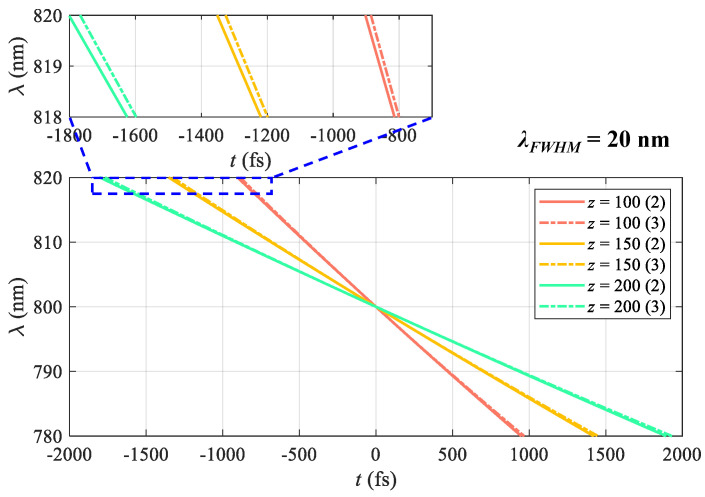
Spectrum–time mapping curves with $\lambda_{0}=800$ nm, $\lambda_{FWHM}=20$ nm, and SF10 glass lengths *z* = [100, 150, 200] mm in red, yellow, and green. Labels (2) and (3) in the legend represent the second and third dispersion orders considered (solid and dashed).

For example, Figure 2 shows the spectrum–time mapping curves with $\lambda_{0}=800$ nm, $\lambda_{FWHM}=20$ nm, and SF10 glass lengths *z* = [100, 150, 200] mm in red, yellow, and green, respectively. The influence of the high-order dispersion is also explored. The high-order terms in Equation (5) are labeled in the legend, with (2) and (3) representing the second and third orders (solid and dashed lines). The high-order dispersion slightly affects the spectrum–time mapping model, as shown in the zoomed-in figure labeled with the blue box. If the high-order dispersion is not negligible, we need to calibrate the time labels of the spectrally resolved frames with the mapping model.

### 2.2. Evaluation Criteria

**Figure 3:**
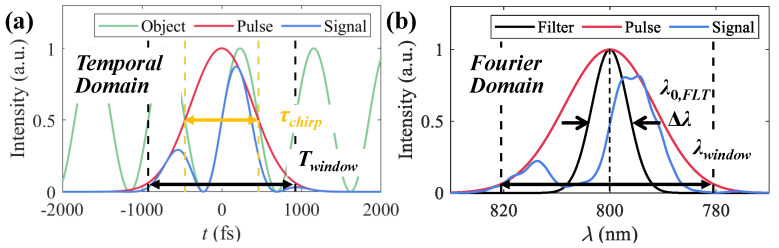
(**a**) Object’s information (green), illumination pulse (red), and modulated signal (blue) in temporal domain. (**b**) Spectral filter (black), illumination pulse (red), and modulated signal (blue) in Fourier domain.

For simplification, one-dimensional detection using chirped pulses is demonstrated. The object’s transmission function $I_{obj}$ is simulated as a sine function with a period of $T_{s}$ and is assumed to be homogeneous over the spectrum of the chirped illumination, as shown in Figure 3a (green),

(14) $$I_{obj}=\left[1+\sin\left(2\pi T/T_{s}\right)\right]/2$$

The signal (Figure 3a, blue) after object modulation is

(15) $$I_{sig}=I_{chirp}I_{obj}$$

The corresponding Fourier domain distributions of $I_{chirp}$ and $I_{sig}$ are shown in Figure 3b as the red and blue lines. In the comparison of the chirped pulses, they precede and postdate the object’s modulation; diverse wavelengths carry the information from different moments of the object. Filters are used for time-resolved signal extraction, whose spectrum curves are assumed to follow Gaussian functions $FLT(\lambda_{0,\;FLT},\Delta \lambda )$ with a center wavelength of $\lambda_{0,FLT}$ and a FWHM of Δλ, as shown in Figure 3b (black). By scanning FLT in the observing window $\lambda_{window}$ in the Fourier domain with a step of $\lambda_{interval}$, multiple frames can be extracted.

The filtered object signal ${\tilde{E}}_{sig,\;\;FLT}$ and reference signal ${\tilde{E}}_{ref,\;\;FLT}$ in the Fourier domain can be expressed as

(16) $${\tilde{E}}_{sig,\;\;FLT}={\tilde{E}}_{sig}FLT\left(\lambda_{0,\;FLT},\Delta \lambda \right)$$

(17) $${\tilde{E}}_{ref,\;\;FLT}={\tilde{E}}_{chirp}FLT\left(\lambda_{0,\;FLT},\Delta \lambda \right)$$

$E_{sig,\;\;FLT}$ and $E_{ref,\;\;FLT}$ in the temporal domain can be derived with the inverse Fourier transform. $I_{sig,\;\;FLT}\propto {\left|E_{sig,\;\;FLT}\right|}^{2}$ is calibrated by dividing $I_{ref,\;\;FLT}\propto {\left|E_{ref,\;\;FLT}\right|}^{2}$ to eliminate the influence of illumination’s non-uniform intensity distribution. $I_{sig,\;\;FLT}$ is integrated temporally by a detector to output a measurement ${\tilde{I}}_{mrs}$ at $\left(\lambda_{0,\;FLT},\Delta \lambda \right)$. Likewise, ${\tilde{I}}_{mrs}$ is calibrated by dividing ${\tilde{I}}_{ref}$, which is the integration of $I_{ref,\;\;FLT}$.

By scanning the filter over the spectrum, ${\tilde{I}}_{mrs}$ at different $\lambda_{0,FLT}$ is obtained. According to the relationship between spectrum and time, the temporal domain $I_{msr}$ is derived. The length of $I_{msr}$ is the frame number $N_{frame}$, and the time interval between adjacent measurements is the frame interval whose multiplicative inverse is the frame rate.

The time resolution depends on the similarity between $I_{msr}$ and $I_{obj}$, which is evaluated with two parameters in this work, the relative modulation *M* and similarity index *R*,

(18) $$M=M_{mrs}/M_{0},M_{mrs}=\frac{I_{mrs,\;max}-I_{mrs,\;min}}{I_{mrs,\;max}+I_{mrs,\;min}},M_{0}=\frac{I_{obj,\;max}-I_{obj,\;min}}{I_{obj,\;max}+I_{obj,\;min}}$$

(19) $$MSE=\frac{1}{N_{frame}}\sqrt{\sum {\left(I_{mrs}-I_{obj}\right)}^{2}},R=\frac{MSE_{max}-MSE}{MSE_{max}}$$

The modulation of $I_{obj}$ is set to $M_{0}=1$, with $I_{obj,\;max}=1$ and $I_{obj,\;min}=0$. $M_{mrs}$ is the modulation of $I_{mrs}$. *M* indicates the capability of resolving two adjacent peaks, while *R* represents the degree of measurement distortion. MSE is the mean squared error between $I_{obj}$ and $I_{mrs}$. $MSE_{max}$ is the MSE when the measurement is completely flat ($I_{mrs}$ = 0.5) without time-resolved information. If $MSE>MSE_{max}$, $I_{msr}$ is heavily distorted with a non-negligible phase change from $I_{obj}$. Therefore, to ensure a high-fidelity time-resolved measurement, *M* and *R* should simultaneously satisfy some criteria to determine the minimum resolvable period $T_{s,min}$ of $I_{obj}$. $T_{s,min}$ indicates the time resolution. The criteria for determining $T_{s,min}$ are M>10% and R>10% in this work.

The valid time observing window is defined as $T_{window}=\left[-\tau_{chirp},\tau_{chirp}\right]$, as shown in Figure 3. The maximum period of $I_{obj}$ that is available for evaluation is set to $T_{s,max}=\tau_{chirp}$. Poisson noise is considered, and the number of photons of the illumination pulse is denoted as $N_{p}$ in the following simulations.

## 3. Temporal Characteristics

**Figure 4:**
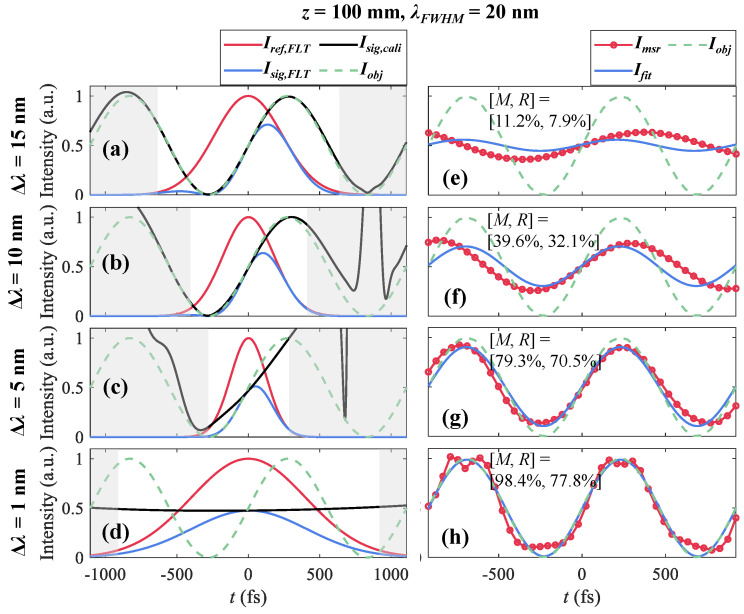
Filtered reference signal $I_{ref,FLT}$ (red), object signal $I_{sig,FLT}$ (blue), and calibrated signal $I_{sig,cali}=I_{sig,FLT}/I_{ref,FLT}$ (black) using filters with $\lambda_{0,FLT}=\lambda_{0}$ and Δλ = (**a**) 15 nm, (**b**) 10 nm, (**c**) 5 nm, and (**d**) 1 nm. For Δλ = (**e**) 15 nm, (**f**) 10 nm, (**g**) 5 nm, and (**h**) 1 nm, measurements $I_{mrs}$ (red) and fitted results $I_{fit}$ (blue) with the shifting of $\lambda_{0,FLT}$ in $\lambda_{window}$ with 20 sample points in a period $T_{s}$. Ground truth $I_{obj}$ ($T_{s}$ = 400 fs, green dotted). The gray shaded area represents the temporal area of the filtered pulse with the relative intensity lower than 0.1.

The influence of the filtering bandwidth Δλ on the underlying changes of the chirped pulse and the measurements is demonstrated in Figure 4. The ground truth of $I_{obj}$ ($T_{s}$ = 400 fs) is represented as the green dotted line. SF10 glass rods with lengths *z* are adopted for generating chirped pulses for illumination in the following simulations. A bandwidth-limited pulse with $\lambda_{FWHM}$ = 20 nm ($\tau_{0}$ = 47 fs) and $N_{p}=10^{4}$ at $\lambda_{0}$ = 800 nm is broadened into a chirped pulse ($\tau_{chirp}$ = 923 fs) with *z* = 100 mm.

Figure 4a–d show the filtered reference signal $I_{ref,FLT}$ (red), filtered object signal $I_{sig,FLT}$ (blue), and calibrated signal $I_{sig,cali}=I_{sig,FLT}/I_{ref,FLT}$ (black) using filters with $\lambda_{0,FLT}=\lambda_{0}$ and Δλ = 15 nm, 10 nm, 5 nm, and 1 nm, respectively.

The measured results $I_{mrs}$ are produced by integrating $I_{sig,FLT}$ and $I_{ref,FLT}$ temporally at a series of $\lambda_{0,FLT}$. $I_{mrs}$ is shown in red with circular markers and is fitted with a sine function $I_{fit}$ with a period of $T_{s}$ in Figure 4e–h. With a large Δλ = 15 nm, $I_{sig,FLT}$ contains plenty of the object’s information and $I_{sig,cali}$ (black) highly conforms to $I_{obj}$ in Figure 4a. $I_{mrs}$, shown in Figure 4e, is a complanate curve due to each spectrally resolved measurement covering similar object information, leading to unresolvable peaks of $I_{obj}$.

While dispersion plays a domain role on filtered pulse widths, narrower $I_{sig,FLT}$ and $I_{ref,FLT}$ are produced with decreased Δλ, as shown in Figure 4b,c. Meanwhile, less object information is involved in $I_{sig,FLT}$. $I_{sig,cali}$ is similar to the ground truth in a limited range and seems to be stretched, as the slope is flatter than $I_{obj}$ around *t* = 0 fs. $I_{mrs}$ varies as different filters cover different object information, as shown in Figure 4f,g. The evaluative parameters [M,R]=[39.6%,32.1%] for Δλ = 10 nm and [M,R]=[79.3%,70.5%] for Δλ = 5 nm.

As Δλ decreases, the Fourier transform limits filtered pulse widths, which can be much wider than the chirped pulse. However, $I_{sig,FLT}$ does not contain more object information covered by the filtered pulse. The valid object’s information contained is still in a limited range and is stretched over the filtered pulse, as shown in Figure 4d. The performance of $I_{mrs}$ is improved with [M,R]=[98.4%,77.8%].

**Figure 5:**
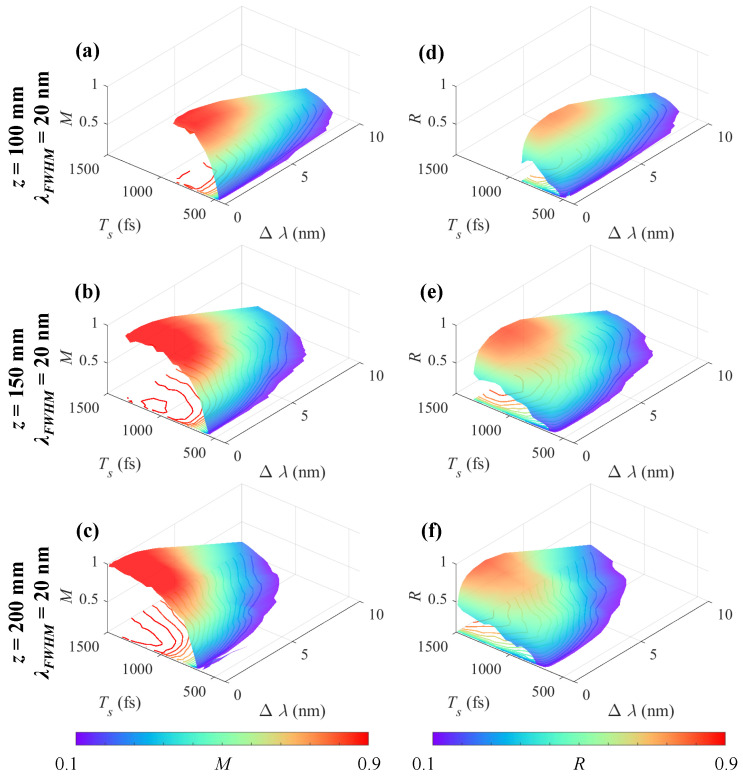
Measurement performance for $I_{obj}$ with different $T_{s}$ and Δλ using chirped pulses ($\lambda_{FWHM}$ = 20 nm and $N_{p}=10^{4}$). (**a**–**c**) *M* and (**d**–**f**) *R* diagrams for *z* = 100 mm, 150 mm, and 200 mm.

Figure 5 shows the influence of $T_{s}$ and Δλ on the measurement performance of $I_{obj}$ using chirped pulse illumination ($\lambda_{FWHM}$ = 20 nm and $N_{p}=10^{4}$).

Figure 5a–c show *M* diagrams and Figure 5d–f show *R* diagrams for *z* = 100 mm, 150 mm, and 200 mm with $\tau_{chirp}$ = 923, 1384, and 1845 fs. The lower bounds of $T_{s}$ that can be resolved increase with increasing *z* or Δλ. For a fixed $T_{s}$, a smaller Δλ leads to a lower signal-to-noise ratio (SNR) due to Poisson noise, while a larger Δλ decreases the ability to extract spectrally resolved information from $I_{obj}$. Therefore, there is an optimum Δλ leading to maximum *M* and *R*.

**Figure 6:**
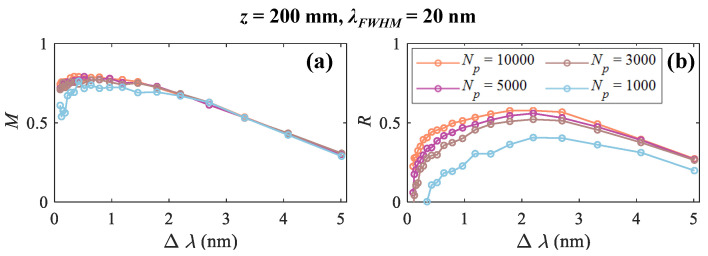
(**a**) *M* and (**b**) *R* curves with $N_{p}=\left[10^{4},5\times 10^{3},3\times 10^{3},10^{3}\right]$ in orange, carmine, brown, and blue for the case $\lambda_{FWHM}=$ 20 nm and *z* = 200 mm.

The influence of $N_{p}$ on measurement performance is shown in Figure 6 for the case $\lambda_{FWHM}=$ 20 nm and *z* = 200 mm. *M* and *R* curves with $N_{p}=\left[10^{4},5\times 10^{3},3\times 10^{3},10^{3}\right]$ are in orange, purple, brown, and blue, respectively. The similarity index *R* is more sensitive to $N_{p}$. *M* and *R* have maximum values around 0.5 nm and 2.2 nm, respectively. Therefore, Δλ in the range of [0.5, 2.2] nm can be set to make a compromise between *M* and *R*. The lower the SNR, the larger the Δλ should be selected. In the following simulations, cases with $N_{p}=10^{4}$ are considered.

**Figure 7:**
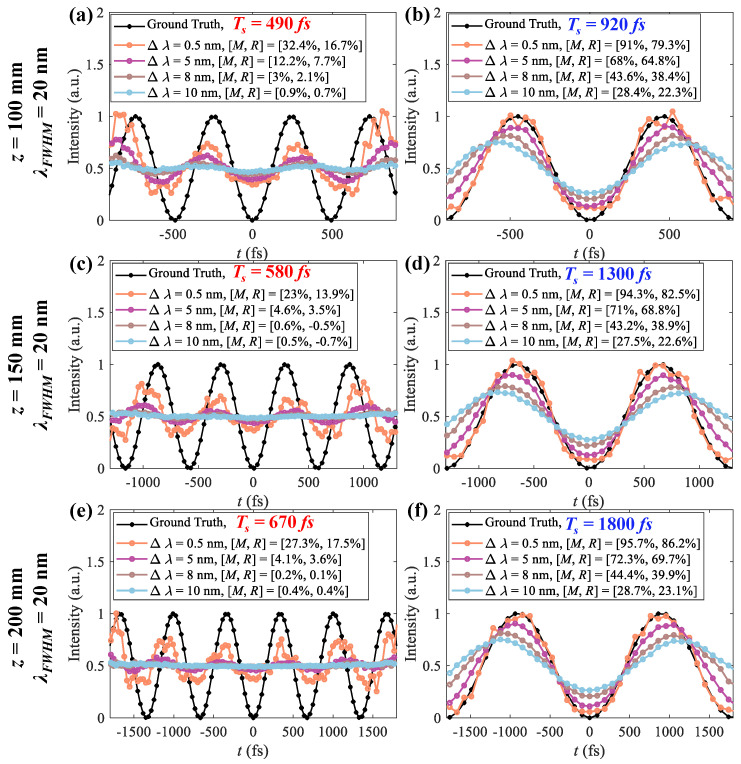
Measurements $I_{msr}$ of $I_{obj}$ (black) around the lower and upper bounds: (**a**,**b**) *z* = 100 mm, (**c**,**d**) *z* = 150 mm, and (**e**,**f**) *z* = 200 mm and Δλ = 0.5 nm (orange), 5 nm (purple), 8 nm (brown), and 10 nm (blue).

Figure 7 shows the measurements $I_{msr}$ of $I_{obj}$ (black) with different Δλ around the lower bounds $T_{s}$ = 490 fs, 580 fs, and 670 fs and the upper bounds $T_{s}$ = 920 fs, 1300 fs, and 1800 fs for *z* = 100 mm, 150 mm, and 200 mm. $I_{msr}$ with Δλ = 0.5 nm, 5 nm, 8 nm, and 10 nm is in orange, purple, brown, and blue, respectively. On the lower bounds, $I_{msr}$ with Δλ = 0.5 nm satisfies the criteria for time resolution (*M* > 0.1 and *R* > 0.1), whereas the other cases are failed. On the upper bounds, two peaks can be resolved for all cases, and $I_{mrs}$ have lower *M* and *R* with a larger Δλ. Consequently, the time resolution and time window are mutually restricted, and larger Δλ leads to degraded measurements.

**Figure 8:**
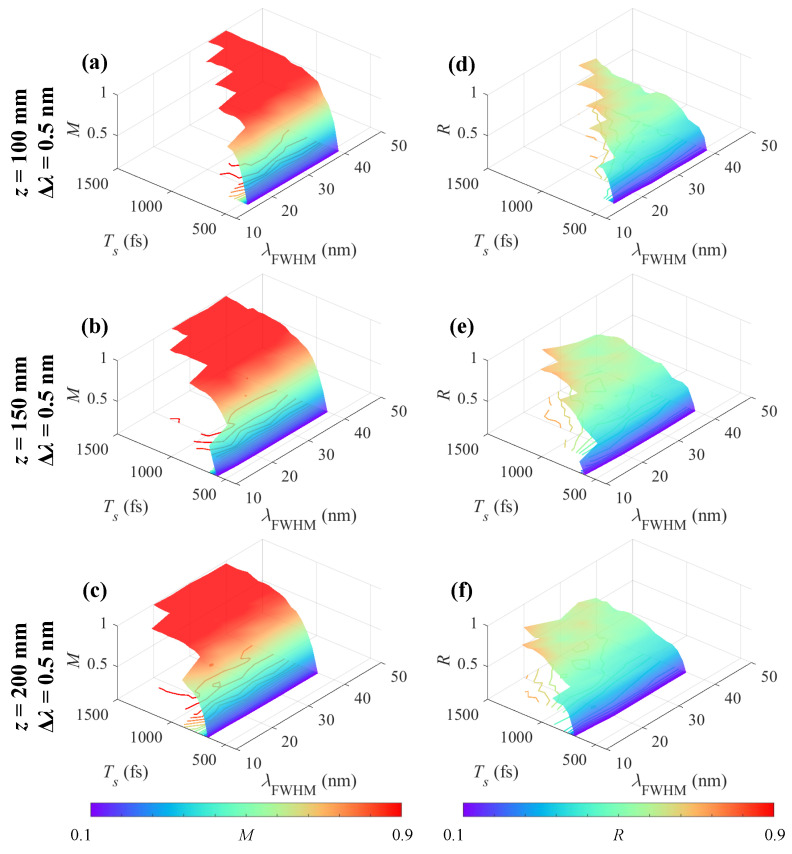
Measurement performance for $I_{obj}$ using chirped pulses with different $T_{s}$ and $\lambda_{FWHM}$. (**a**–**c**) *M* and (**d**–**f**) *R* diagrams for *z* = 100 mm, 150 mm, and 200 mm. Δλ = 0.5 nm.

Figure 8 shows the influence of $T_{s}$ and $\lambda_{FWHM}$ on the measurement performance of $I_{obj}$ using Δλ = 0.5 nm. Figure 8a–c show *M* diagrams, and Figure 8d–f show *R* diagrams for *z* = 100 mm, 150 mm, and 200 mm, respectively. The upper bound $T_{s,max}$ increases gradually with larger $\lambda_{FWHM}$ because of a longer pulse duration. The lower bound $T_{s,min}$ is insensitive to $\lambda_{FWHM}$ when Δλ and *z* is fixed, but sensitive to *z*.

**Figure 9:**
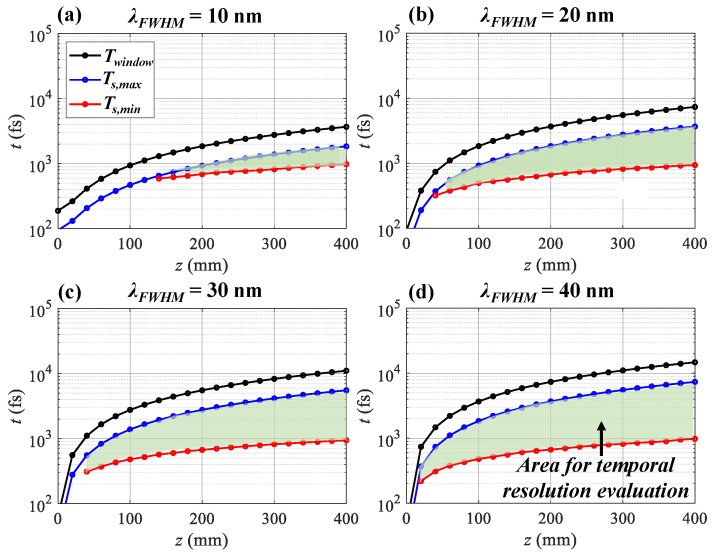
Time window $T_{window}$, lower and upper bounds ($T_{s,min}$, $T_{s,max}$) of the time resolution evaluation range depending on *z* for $\lambda_{FWHM}$ = (**a**) 10 nm, (**b**) 20 nm, (**c**) 30 nm, and (**d**) 40 nm.

To explore the influence of $\lambda_{FWHM}$ further, the lower and upper bounds ($T_{s,min}$, $T_{s,max}$) depending on *z* with $\lambda_{FWHM}$ = 10 nm, 20 nm, 30 nm, and 40 nm are shown in Figure 9. $T_{s,min}$ is determined with the criteria *M* > 0.1 and *R* > 0.1. Δλ = 0.5 nm for all cases. The green area limited by the $T_{s,min}$ and $T_{s,max}$ curves is the evaluation area for time resolution. When $\lambda_{FWHM}$ = 10 nm, the pulse is stretched from 94 fs to 742 fs to provide a minimum $T_{s,min}$ = 590 fs at *z* = 140 mm (Figure 9a). With larger $\lambda_{FWHM}$, $T_{s,min}$ can be pushed lower $T_{s,min}$ = 320 fs @ *z* = 40 mm, 310 fs @ *z* = 40 mm, and 220 fs @ *z* = 20 mm for $\lambda_{FWHM}$ = 20 nm, 30 nm, and 40 nm. With the increase in *z*, the pulse is stretched broader, leading to a wider $T_{window}$. Meanwhile, the time resolution degrades. At *z* = 400 mm, $T_{s,min}$ = 980 fs for $\lambda_{FWHM}$ = 10 nm, 20 nm, 30 nm, and 40 nm.

## 4. Discussion of Evaluation Methods

### 4.1. Theoretical Evaluation

**Figure 10:**
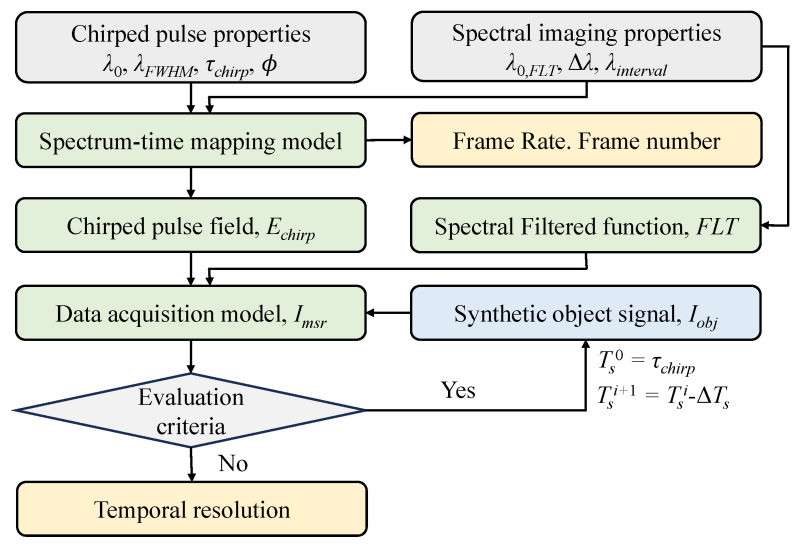
Diagram of the theoretical evaluation procedure.

The theoretical evaluation procedure is depicted in Figure 10. The properties of the chirped illumination pulse and the spectral imaging module are needed prior to the theoretical analysis, which can be measured experimentally. $\lambda_{0}$, $\lambda_{FWHM}$, $\tau_{chirp}$, and ϕ are used to build the spectrum–time mapping model T(λ), whose slope is denoted as S=|dT/dλ|. $\lambda_{0,FLT}$, Δλ, and $\lambda_{interval}$ are calibrated for a particular spectral imaging module. The frame rate depends on *S*, $FR=1/S\lambda_{interval}$. The frame number can be derived as $N_{frame}=\lambda_{window}/\lambda_{interval}$.

For the temporal resolution, synthetic object signals $I_{obj}$ following sine functions with a period of $T_{s}$ are adopted to simulate the data acquisition process. Considering the expressions of chirped pulse $E_{chirp}$ and the spectral filter FLT, the data acquisition model can provide the measured signal $I_{msr}$ for $I_{obj}$ with a period $T_{s}$. $I_{msr}$ is evaluated with the criteria of the relative modulation *M* > 0.1 and similarity index *R* > 0.1. By iteratively decreasing $T_{s}$ ($T_{s}^{0}=\tau_{chirp}$, $T_{s}^{i+1}=T_{s}^{i}+\Delta T$, *i* = 1, 2, 3, …, ΔT is the step), the temporal resolution is determined as $T_{s}^{i-1}$ when the criteria are not satisfied at $T_{s}^{i}$.

### 4.2. Experimental Evaluation

For ultrafast imaging methods based on active pulsed illumination, the difficulty in experimental evaluation is the construction of a standard dynamic process to be detected. To address the issue, an optical Kerr gate (OKG) using high-refractive solid glasses can be used with a response time on the scale of 100 fs [23,24].

**Figure 11:**
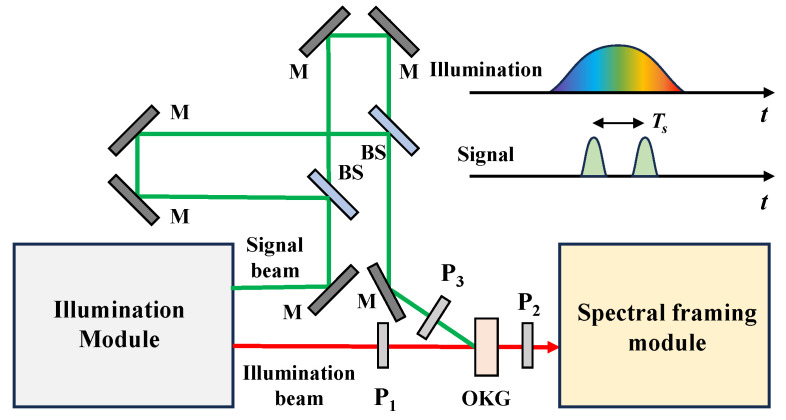
Diagram of the potential experimental evaluation setup. The signal and illumination beams are in green and red, respectively. BS: beam splitter, M: mirror, P: polarizer, OKG: optical Kerr gate.

Figure 11 shows the diagram of the potential experimental evaluation setup. The illumination module outputs a chirped illumination beam and a non-chirped signal beam, which are synchronized on the Kerr medium. The signal beam contains two sub-pulses whose interval ($T_{s}$) can be adjusted with delay lines and whose polarizations are 45° after the polarizer P_3_. The temporal variation of the refractive index ellipsoid is the convolution of the signal beam and the Kerr medium’s response. The illumination beam probes the induced transient birefringence by the polarization detecting through a pair of crossed polarizers (P_1_ and P_2_) around the Kerr medium.

The key point is to construct a dynamic refraction index change with two resolvable peaks whose interval is $T_{s}$. However, this strategy is limited due to the relaxation time of the OKG $t_{OKG}$. To ensure the two peaks induced by the two sub-pulses in the signal beam, $T_{s}\gg t_{OKG}$ should be satisfied. The measured results with the evaluated ultrafast imaging system should resolve the two peaks when the temporal resolution is better than $T_{s}$.

## 5. Conclusions

For ultrafast imaging methods based on continuous chirped pulse illumination, the temporal characteristics are investigated in our research, promoting the further analysis of targets’ dynamic behavior. The main factors that affect the temporal resolution are the illumination pulse’s bandwidth $\lambda_{FWHM}$, dispersive degree of the pulse (lengths *z* of SF10 glass rods in simulations), and spectral resolution Δλ of the framing module. To demonstrate the influence quantitatively, a time-resolved sinusoidal signal with a period of $T_{s}$ is used. The minimum period $T_{s,min}$ of the resolvable sinusoidal signal is interpreted as the time resolution under the criteria determining the similarity of the measurement and ground truth.

The time window and time resolution are quantitatively analyzed. With the increase in $\lambda_{FWHM}$ and *z*, $T_{window}$ becomes broader. Larger *z* and Δλ reduce the time resolution. However, *z* must be long enough to cover a period that can be resolved, and Δλ must be broader enough to include more photons to avoid distortion due to a low SNR. This work provides theoretical and experimental procedures to determine and optimize the temporal resolving capabilities of a continuous chirped pulse illuminated ultrafast imaging system, supporting the precise characterization of dynamic processes. Potential experimental evaluation strategies will be explored in the future.

## Data Availability

The raw data supporting the conclusions of this article will be made available by the authors on request.
